# Transport-of-intensity phase imaging using commercially available confocal microscope

**DOI:** 10.1117/1.JBO.29.11.116002

**Published:** 2024-11-07

**Authors:** Naru Yoneda, Joe Sakamoto, Takumi Tomoi, Tomomi Nemoto, Yosuke Tamada, Osamu Matoba

**Affiliations:** a Kobe University, Graduate School of System Informatics, Department of System Science, Kobe, Japan; b Kobe University, Center of Optical Scattering Image Science, Kobe, Japan; c Exploratory Research Center on Life and Living Systems, Biophotonics Research Group, Okazaki, Japan; d National Institute for Physiological Sciences, Division of Biophotonics, Okazaki, Japan; e Utsunomiya University, Faculty of Engineering, Utsunomiya, Japan; f Utsunomiya University, Institute for Social Innovation and Cooperation, Center for Innovation Support, Utsunomiya, Japan; g Tokyo University of Science, Department of Applied Biological Science, Faculty of Science and Technology, Noda, Japan; h The Graduate University for Advanced Studies (SOKENDAI), School of Life Science, Okazaki, Japan; i Utsunomiya University, Center for Optical Research and Education, Utsunomiya, Japan; j Utsunomiya University, Robotics, Engineering and Agriculture-Technology Laboratory, Utsunomiya, Japan

**Keywords:** quantitative phase imaging, transport of intensity equation, microscopy, confocal microscopy, fluorescence imaging, fluorescence microscopy

## Abstract

**Significance:**

Confocal microscopy is an indispensable tool for biologists to observe samples and is useful for fluorescence imaging of living cells with high spatial resolution. Recently, phase information induced by the sample has been attracting attention because of its applicability such as the measurability of physical parameters and wavefront compensation. However, commercially available confocal microscopy has no phase imaging function.

**Aim:**

We repurpose an off-the-shelf confocal microscope as a phase measurement microscope. This is a milestone in changing the perspective of researchers in this field. We would meet the demand of biologists if only they had measured the phase with their handheld microscopes.

**Approach:**

We proposed phase imaging based on the transport of intensity equation (TIE) in commercially available confocal microscopy. The proposed method requires no modification using a bright field imaging module of a commercially available confocal microscope.

**Results:**

The feasibility of the proposed method is confirmed by evaluating the phase difference of a microlens array and living cells of the moss *Physcomitrium patens* and living mammalian cultured cells. In addition, multi-modal imaging of fluorescence and phase information is demonstrated.

**Conclusions:**

TIE-based quantitative phase imaging (QPI) using commercially available confocal microscopy is proposed. We evaluated the feasibility of the proposed method by measuring the microlens array, plant, and mammalian cultured cells. The experimental result indicates that QPI can be realized in commercially available confocal microscopy using the TIE technique. This method will be useful for measuring dry mass, viscosity, and temperature of cells and for correcting phase fluctuation to cancel aberration and scattering caused by an object.

## 1 Introduction

Phase distributions induced by objects cannot directly measure a camera because an image sensor can measure only intensity distributions. Phase-contrast^1^ and differential interference contrast^2^ techniques are proposed for visualizing the phase distributions qualitatively. To quantitatively measure the phase distribution, various quantitative phase imaging (QPI) techniques are proposed^3^ because quantitative phase distribution has informative physical parameters such as optical path difference,^4^ dry mass,^5^ aberrations,^6^ and shear stress.^7^ Numerous QPI techniques have been proposed, and they can be categorized as interferometric or non-interferometric methods. Although interferometric techniques accurately measure the phase distribution, special optical instruments are required to obtain interferograms such as a laser light source, phase-shifter, and interferometry.^8^ Contrarily, non-interferometric methods such as transport-of-intensity equation (TIE)^9^ and iterative phase retrieval algorithms^10^^,^^11^ require no special device because these methods obtain phase distributions using defocused intensity distributions. Particularly, TIE-based QPI can deterministically obtain phase distributions induced by objects. Due to the simplicity of the setup and the algorithm, TIE-based QPI has been applied to various research fields such as low-photon conditions,^12^^,^^13^ fluorescence imaging,^14^ and optical memory.^15^^,^^16^ To improve phase reconstruction accuracy, there are many phase reconstruction algorithms are proposed.^17^^–^^19^ Although TIE-based QPI needs multiple defocused intensity distributions to obtain high-quality phase distributions by shifting an image sensor along an optical axis, various configurations have been proposed to mitigate this problem.^20^^–^^24^ As an advantage of TIE-based QPI, the partially coherent light source can be used for phase imaging.^25^^,^^26^ Although using a partially coherent light source can reduce the speckle noise compared with using a laser, the ambiguity of the phase for wavelength increases when the temporal coherence is low.^27^

TIE-based QPI has been applied to microscopy so far.^28^^,^^29^ In the research field of biology, a confocal laser scanning microscope is usually used for visualizing fluorescence signals from biosamples in real time. To simultaneously measure fluorescence signals and phase distributions, multi-modal microscopy is required. Although the holographic approach measures phase and fluorescence distributions simultaneously,^30^ a specially designed optical setup is required.^31^ While a commercially available wide-field microscope would also be useful for measuring phase and fluorescence simultaneously, the axial resolution of the fluorescence image is degraded. There is an attempt to apply TIE to confocal microscopy;^32^ however, a special varifocal lens is needed to get multiple defocused intensity distributions.

In this paper, we propose TIE-based QPI using commercially available confocal microscopy. Using the bright-field module of the commercially available confocal microscope, we have successfully demonstrated that phase retrieval can be achieved without additional optical elements and specially designed equipment. Because this setup is laser scanning microscopy, the light source is temporally coherent and spatially partially coherent,^33^^,^^34^ which means that detected intensity distributions by the proposed method are speckle-free and the ambiguity of the phase for wavelength is low. We demonstrate phase imaging using living cells of the moss *Physcomitrium patens* and HeLa cells. We also show experimental results of multi-modal fluorescence and phase imaging of living cells under confocal microscopy.

## 2 Methods

**Fig. 1:**
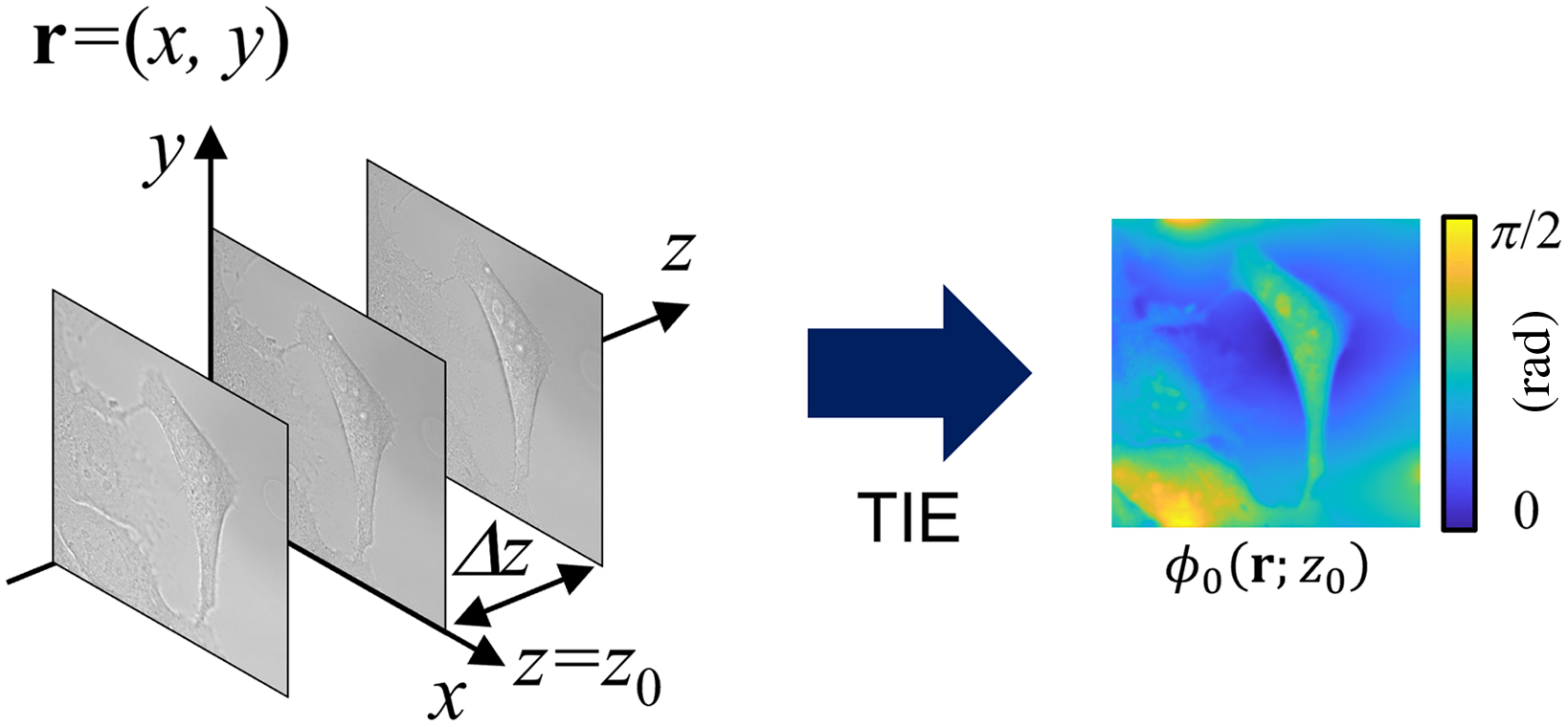
Schematic of the TIE-based phase retrieval.

The schematic of TIE-based QPI is shown in Fig. 1. The TIE is derived from the Helmholtz equation under the condition of paraxial approximation and is described as follows:^9^

$$\nabla_{⊥}\cdot \{I(r;z_{0})\nabla_{⊥}\phi (r;z_{0})\}=-k\frac{\partial I(r;z_{0})}{\partial z},$$ (1)

where $\nabla_{⊥}$ and k denote the gradient operator in the lateral dimension r and the wavenumber, respectively. $\phi (r;z_{0})$ and $I(r;z_{0})$ indicate the phase and intensity distributions of an object at $z=z_{0}$. To solve Eq. (1) for $\phi (r;z_{0})$, the calculation of an axial intensity derivative is required.

In general, the axial intensity derivative is approximated with multiple defocused intensity distributions and is described as follows:^35^

$$\frac{\partial I(r;z_{0})}{\partial z}\approx \sum_{j=-n}^{n}\frac{a_{j}I(r;j\Delta z)}{\Delta z},$$ (2)

where j=−n,…0,…,n. $a_{j}$ is the coefficients of I(r;jΔz). Δz shows a defocus interval. When a target is a pure phase object (phase-only object), the phase distribution can be deterministically obtained using the Fourier transform as follows:^36^

$$\phi (r;z_{0})=-k\mathrm{IFT}[\frac{1}{4\pi^{2}|u{|}^{2}+\alpha }\mathrm{FT}[\nabla_{⊥}\cdot \frac{\nabla_{⊥}}{I(r;z_{0})}\;\times \mathrm{IFT}[\frac{1}{4\pi^{2}|u{|}^{2}+\alpha }\mathrm{FT}[\frac{\partial I(r;z_{0})}{\partial z}]]]],$$ (3)

where FT[…] and IFT[…] are Fourier and inverse Fourier transform operators. u and α are the 2D coordinates of spatial frequencies and a regularization parameter to prevent divergence. $I_{0}$ is an infocus intensity distribution.

## 3 Experiments

**Fig. 2:**
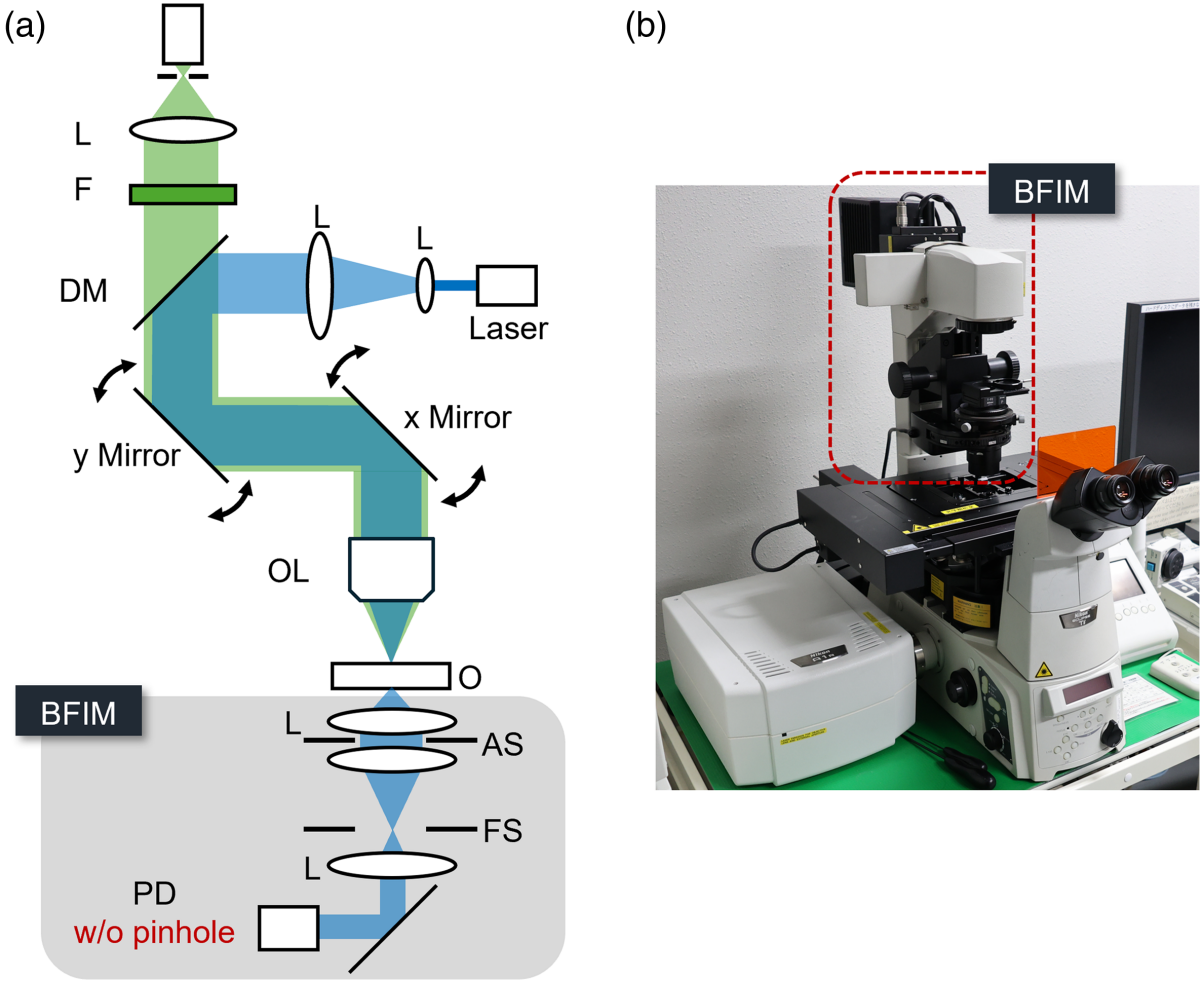
(a) Schematic of an optical setup for commercially available confocal microscopy. L, lens; PD, photodetector; DM dichroic mirror; OL, objective lens; F, bandpass filter; AS, aperture stop; FS, field stop; BFIM, bright field imaging module. (b) Photo of a confocal microscope. The area surrounded by the red dashed line indicates BFIM.

The schematic of the optical setup of confocal microscopy for the proposed method is shown in Fig. 2.

This is an epi-fluorescence confocal microscopy. The light from a laser is scanned at an object plane using Galvano mirrors, and the light from the object goes back to the same optical path and is detected by single-pixel detectors. Due to the pinhole in front of the detector, high axial resolution is achieved in fluorescence imaging. On the other hand, the light through the object is detected by a single-pixel detector without a pinhole. Due to the absence of the pinhole, the detected intensity distributions are similar to bright field images. Because laser scanning microscopy can be considered as a spatially partially coherent technique,^33^^,^^34^ speckle-free images are obtained. By changing the axial position of the object, multiple defocused intensity distributions are obtained. Then, these intensity distributions are used for the axial intensity derivative described in Eq. (2). The object phase distribution can be obtained using the intensity derivative through Eq. (3). Notably, in this study, the samples are mechanically shifted according to the optical axis to get defocus distributions. Although the proposed method can also be applied to real-time imaging, the measurable dynamics are limited by the shift speed. In this study, n is set to 1 to reduce the TIE calculation time. The defocus distance in this study is empirically decided to suppress the noise effect. The regularization parameter is also empirically chosen.

**Fig. 3:**
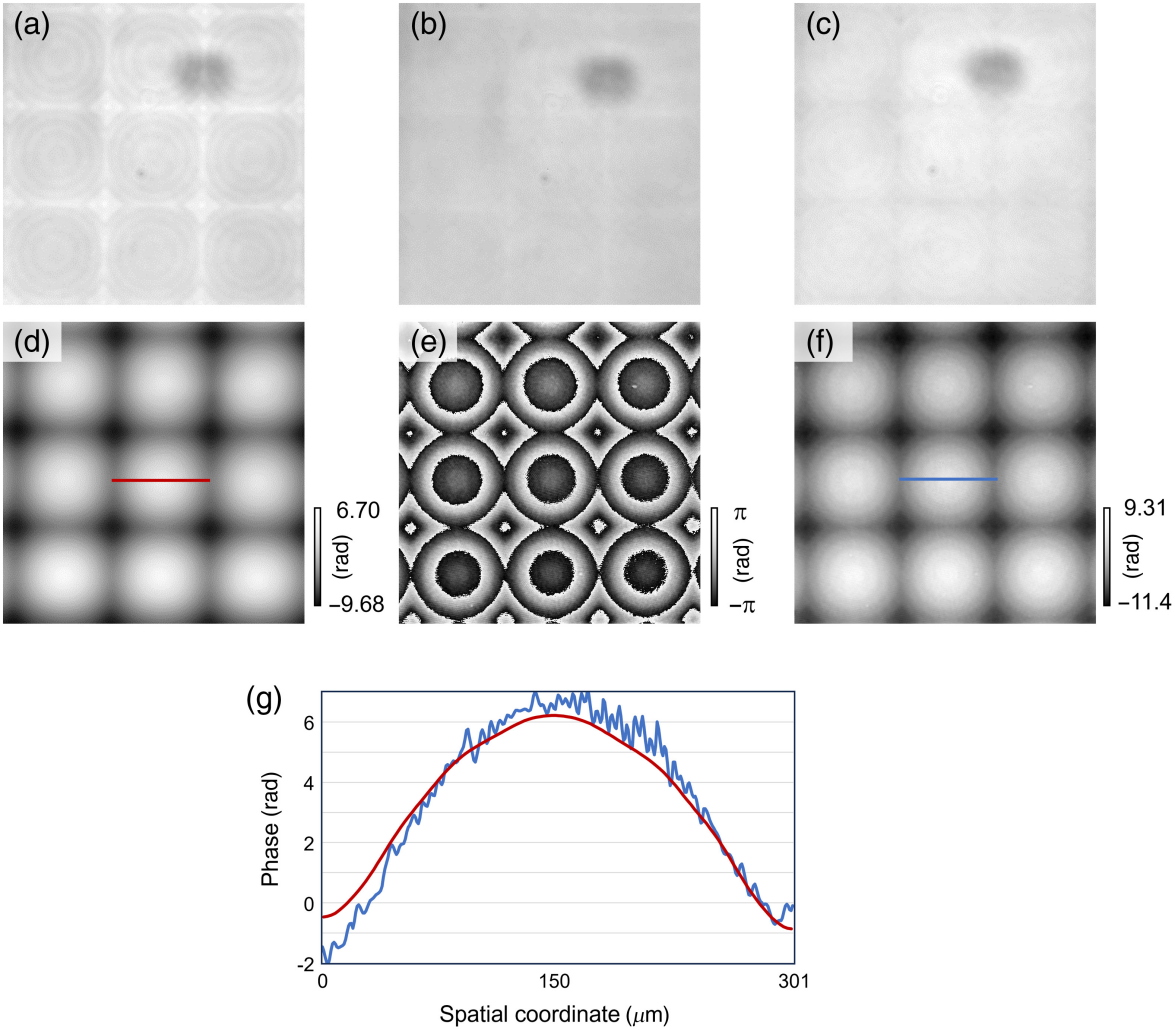
Experimental results of the microlens array, (a)–(c) defocused intensity distributions with the proposed method and (d) phase distributions with the proposed method, (e) wrapped phase with digital holography, (f) unwrapped phase of panel (e), and (g) sectional profiles on red and blue lines.

First, we evaluated the applicability of QPI under the off-the-shelf confocal microscopy using a micro-lens array (Thorlabs, Inc.: MLA300-14AR-M, Newton, United States). The commercially available confocal microscopy (Nikon, Co. Ltd.: A1 Rsi, Tokyo, Japan) was used. The wavelength of the laser was 488 nm. The scanning pitch was set to 2.49  μm. The magnification of the objective lens was 10. The number of pixels was 512×512. The lenslet pitch and the radius curvature of the micro-lens array were 300  μm and 14.2 mm, respectively. The defocused distance for the TIE was set to 100  μm. The measured defocused intensity distributions are shown in Figs. 3(a)–3(c). The phase distribution obtained by the TIE is shown in Fig. 3(d). The micro-lens array was also measured using self-built off-axis digital holographic microscopy with a Mach-Zehnder interferometer. The result of digital holography is shown in Fig. 3(e). The phase distribution obtained by digital holography was unwrapped by the TIE-based phase unwrapping method^37^ and is shown in Fig. 3(f). The sectional profiles at red and blue lines in Figs. 3(d) and 3(e) are shown in Fig. 3(g). To compare the results of Figs. 3(d) and 3(e), bias phases were manually adjusted. The results of the proposed method and digital holography are almost consistent. The experimental results indicate the proposed method can accurately measure the phase distribution of the object.

**Fig. 4:**
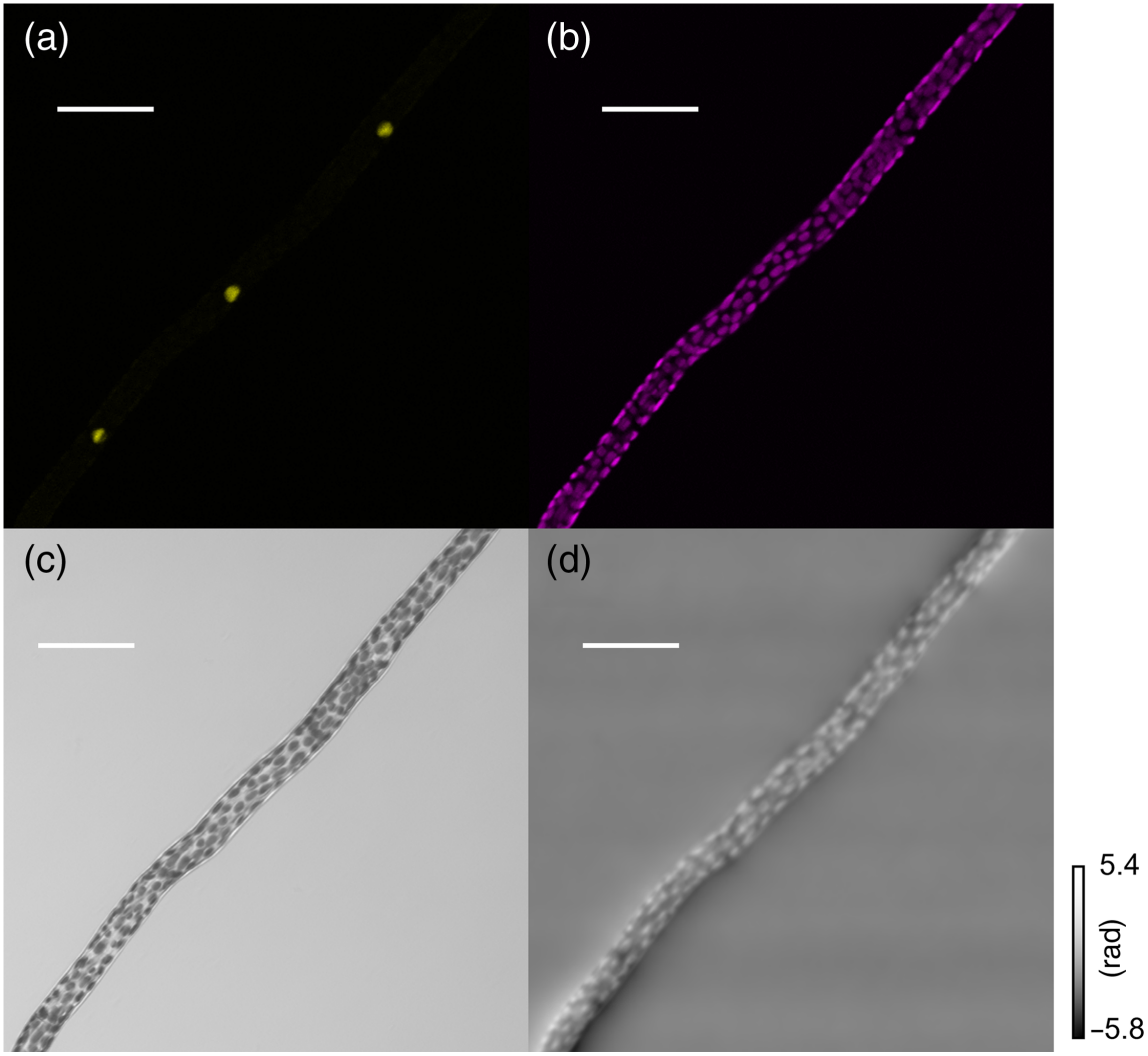
Experimental results of the protonemal cells in *P. patens*, (a) Citrine, (b) chloroplast autofluorescence, (c) bright-field image, and (d) phase distribution. Scale bars represent 50  μm.

Second, we applied the proposed method to visualize the phase distribution of fluorescent protein-labeled living plant cells. We used the filamentous tissue called protonema of the transgenic moss *P. patens*. In the transgenic line, the yellow fluorescent protein gene *Citrine* was inserted into the histone H3.3 locus (Pp3c18_14481),^38^^,^^39^ allowing for detection of fluorescence in the nuclei.^30^ We prepared the samples for confocal microscopy as described previously.^40^ Confocal microscopy (Olympus Co., Ltd.: FV1000, Tokyo, Japan) visualized the plant cells. The wavelength of the laser for phase imaging was 635 nm. The wavelengths of the lasers for exciting the yellow fluorescent protein Citrine localized to the nuclei and chloroplast autofluorescence were 473 and 635 nm, respectively. The scanning pitch was set to 0.621  μm. The images were captured at 0.15 frames per second (fps) and the 3D stack of 41 images took 19 min. The used objective lens was UPLSAPO20X (Olympus). The number of pixels was 1024×1024. The defocused distance for the TIE was set to 3.5  μm. Fluorescent and phase images were simultaneously observed. Experimental results are shown in Fig. 4. The nuclei visualized with Citrine fluorescence in Fig. 4(a) are unidentifiable in the phase distribution of Fig. 4(d), suggesting no clear phase difference distribution between nuclei and cytoplasm. By contrast, chloroplast autofluorescence in Fig. 4(b) highly corresponded to the phase distribution of Fig. 4(d), indicating the large phase difference between chloroplasts and cytoplasm. Compared with chloroplasts in the bright-field image of Fig. 4(c), chloroplast visualized with the phase distribution in Fig. 4(d) shows in-focus chloroplast as Fig. 4(b). This suggests the shallow depth of focus in our method. In Fig. 4(c), the positions of the cross-wall (septa) but not the side-wall are indecisive due to the crowded chloroplasts. However, the phase distribution in Fig. 4(d) improved the visualization of the positions. Our method might facilitate label-free imaging of specific cell walls of plants.

As shown in Fig. 4(c), this sample has absorption, which may affect the phase reconstruction accuracy due to the assumption made in the derivation of Eq. (3). The accuracy of phase measurement in TIE with this moss sample should be compared with that of digital holography in the future. Using a TIE algorithm that takes absorption into account may improve the accuracy of phase measurement.^41^^,^^42^

**Fig. 5:**
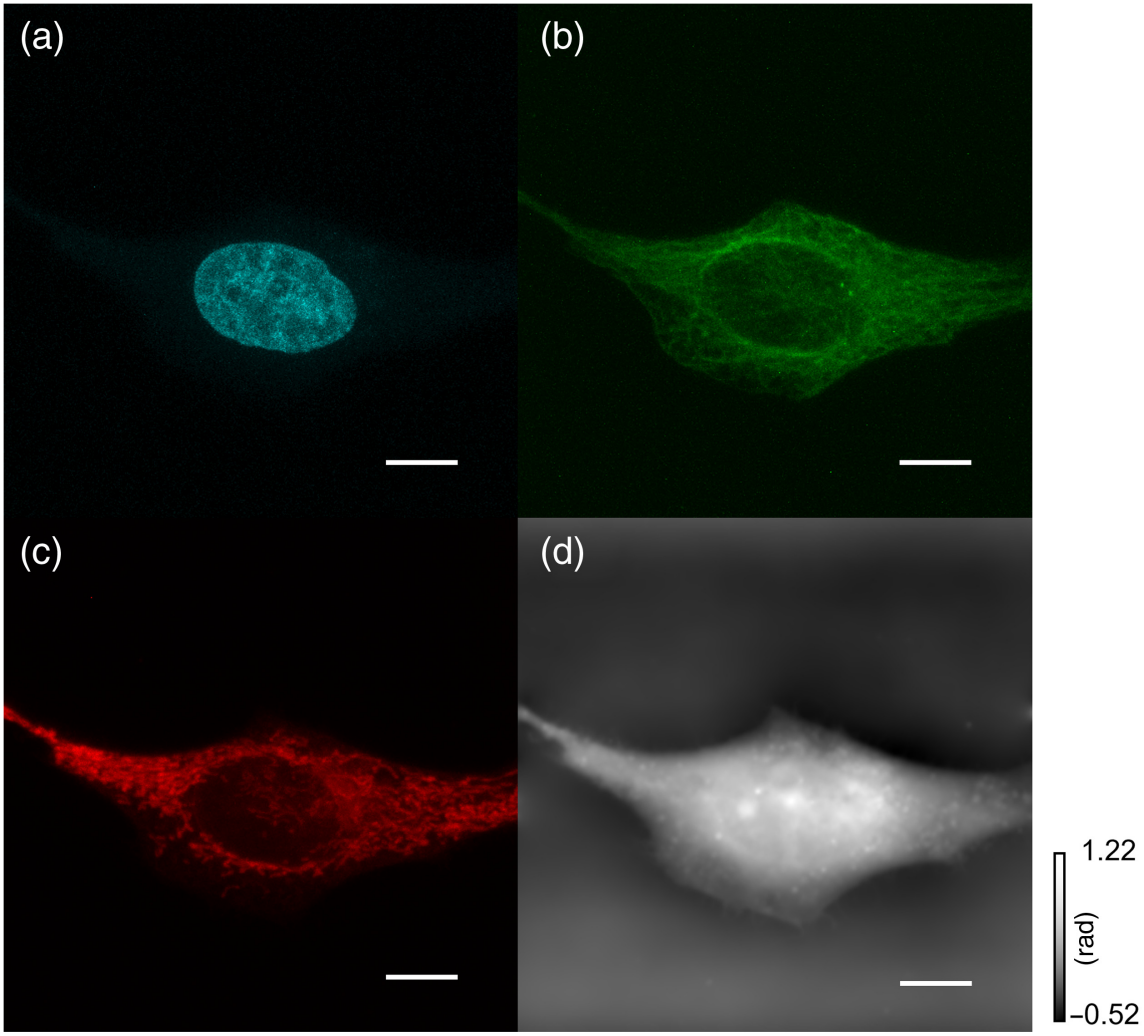
Experimental results of HeLa cell: (a) nucleus, (b) microtubules, (c) mitochondria, and (d) phase distribution. Scale bars represent 10  μm.

Third, we simultaneously observe fluorescence and phase distributions of living mammalian cultured cells, HeLa cells (RCB0007, Riken CellBank, Tsukuba, Japan). Cellular nuclei, mitochondria, and microtubules were stained by NucBlue Live ReadyProbes Reagent (Invitrogen), MitoTracker Red CMXRos, and Tubulin Tracker Green, respectively. Confocal microscopy was performed under the A1 Rsi (Nikon, Co. Ltd.) equipped with a 60× water-immersion objective lens (PlanApo-VC 60xA WI) at room temperature. The transmission light images were obtained using a 488 nm laser and used for TIE-based QPI. The fluorescence images of the nucleus, mitochondria, and microtubules were simultaneously acquired using the excitation laser of 405, 488, and 561 nm. The fluorescent signals of 450±25  nm for nuclei, 525±25  nm for microtubules, and 595±25  nm for mitochondria were detected. The scanning pitch was set to 0.138  μm. The images were captured at 1 fps and the 3D stack of 81 images took 4.7 min. The number of pixels was 512×512. The defocused distance for the TIE was set to 1.25  μm. The fluorescence images of the nucleus, microtubules, and mitochondria are shown in Figs. 5(a)–5(c), and phase distribution is shown in Fig. 5(d). In Fig. 5(d), a curved line-like phase difference was observed around the center of the cell. These phase differences coincide with the edge of the nucleus, nuclear membrane, in Fig. 5(a). Furthermore, circular phase differences in the nuclear region [Fig. 5(d)] correspond to nucleoli visualized as lacking fluorescence in the nucleus [Fig. 5(a)]. This result suggests that the nuclear membrane and nucleoli can be visualized without labeling by the proposed method. Although individual mitochondria clearly visualized in fluorescence image were indistinguishable from the phase distribution, the region with a higher density of mitochondria had relatively higher phase difference from other regions [Figs. 5(a) and 5(d)]. By contrast, microtubules visualized with fluorescent dyes [Fig. 5(b)] were not identifiable in Fig. 5(d). These suggest that no clear phase differences were detected between cytosol and microtubules by the proposed method. Note that the processing time for the TIE calculation is about 0.8 s with an Intel Core i7-11700 CPU at 2.50 GHz.

## 4 Conclusion

In this paper, we proposed TIE-based QPI using commercially available confocal microscopy. We evaluated the feasibility of the proposed method using the microlens array. The experimental result indicates that QPI can be realized in commercially available confocal microscopy using the TIE technique. In addition, we applied this technique to practical cases using plant and mammalian cultured cells. This method will be useful for measuring dry mass,^5^ viscosity, and temperature of cells and for correcting phase fluctuation to cancel aberration and scattering caused by an object.^43^ To obtain information such as dry mass or intracellular temperature distribution, the accuracy of this method, including its sensitivity and reliability, must be ensured. In future work, it will be necessary to compare and evaluate the phase detection sensitivity of the proposed method with other established quantitative phase measurement techniques. The proposed method could be further improved through the fusion of diffraction tomography. One limitation of the proposed method is that the general TIE-based analysis assumes samples with small scattering and absorption effects. Therefore, when the effects of scattering and absorption are dominant, the phase distribution obtained using this method is less accurate than the results obtained by general interferometric methods. This effect could be mitigated by applying a diffraction tomography algorithm^29^ or by combining a reconstruction method that takes absorption effects into account.^41^^,^^42^ An example of an application of confocal microscopy is the surface inspection of substrates. In this paper, we have measured biological samples, but it could be applied to the inspection of objects such as diffractive optical elements.

## Data Availability

All relevant data, materials, and software code used in this research are available upon request from the corresponding author.
